# Clinical efficacy of sertraline in the treatment of depression caused by Alzheimer disease: A protocol of systematic review

**DOI:** 10.1097/MD.0000000000023076

**Published:** 2020-11-06

**Authors:** Wei-hua Li, Zhuo-wen Wei, Xiao-feng Liu

**Affiliations:** a Department of Neurology; b Department of Anesthesiology, Baoji Traditional Chinese Medicine Hospital, Baoji; c Department of Psychiatry, Xi’an Mental Health Center, Xi’an, Shaanxi, China.

**Keywords:** Alzheimer disease, depression, efficacy, sertraline

## Abstract

**Background:**

This study will appraise the clinical efficacy of sertraline in the treatment of depression caused by Alzheimer disease (AD).

**Methods:**

Comprehensive searches in PUBMED, EMBASE, Cochrane Library, Scopus, AMED, CNKI, and WANGFANG will be performed from inception to the present without language restriction. In addition, other sources will also be searched to avoid losing more potential studies. We will only consider randomized controlled trials that examined the efficacy of sertraline for depression in patients with AD. Two team members will independently undertake literature selection, data collection, and risk of bias assessment. We will use Cochrane Risk of Bias Tool to assess the risk of bias for each eligible trial, and will utilize RevMan 5.3 software to carry out data analysis.

**Results:**

This study will recapitulate high-quality evidence to assess the efficacy of sertraline for the treatment of depression following AD.

**Conclusion:**

The findings of this study will help to determine whether or not sertraline is effective for the treatment of depression after AD.

**OSF registration:**

osf.io/f29v6.

## 1 Introduction

Alzheimer disease (AD) is a progressive neurodegenerative disorder,^[1–3]^ which is characterized by memory deficits and cognitive decline.^[4,5]^ It is also the most leading cause of dementia globally.^[6,7]^ Studies reported that this condition affects millions of predominantly elderly individuals worldwide.^[8,9]^ Studies found that it is associated with aging, and it mostly affects individual over 65 years old.^[10–12]^ It is estimated that about 248 million aged people (account for 17.17% of whole population) will suffer from AD in China by 2020.^[13,14]^ Currently, there remain no effective management options for most patients. Thus, patients with AD often experience a couple of psychological issues, such as depression, anxiety.^[15–18]^ Fortunately, a variety of managements are available for the treatment of depression after AD.^[19–21]^ Of those, sertraline is one of the most effective medication for the treatment of depression following AD.^[22–26]^ However, there is no systematic review to investigate this issue. Therefore, the present study will systematically and comprehensively appraise the clinical efficacy of sertraline in the treatment of depression in patients with AD.

## 2 Methods

### 2.1 Study registration

We have registered this study through OSF (osf.io/f29v6). We have organized this protocol based on the guidelines of Preferred Reporting Items for Systematic Reviews and Meta-Analysis Protocol Statement.^[27]^

### 2.2 Ethics and dissemination

No ethic approval is necessary for this study, because it will analyze published available data only. We plan to publish this study on a peer-reviewed journal.

### 2.3 Eligibility criteria for study selection

#### 2.3.1 Types of studies

This study will include randomized controlled trials (RCTs) focusing on the efficacy of sertraline for depression after AD. We will eliminate unqualified studies, such as review, nonclinical trial, uncontrolled trial, and quasi-RCTs.

#### 2.3.2 Types of participants

Studies that enrolled adults (≥18 years old) diagnosed as depression in AD patients will be selected for inclusion. We will exclude patients with depression caused by other reasons, except AD. We will not impose limitations to other factors, such as race and gender.

#### 2.3.3 Types of interventions

##### 2.3.3.1 Intervention

In the intervention group, all patients underwent any forms of sertraline in the treatment of depression after AD.

##### 2.3.3.2 Comparator

In the control group, any therapy can be used as a control treatment, such as medication, Chinese herbal medicine, acupuncture, placebo, or no treatment. We will exclude combined therapy with sertraline.

#### 2.3.4 Type of outcome measurements

Primary outcome is depression (measured by any related tool, such as Hamilton Depression Scale). Secondary outcomes are anxiety, health-related quality of life, and adverse events.

### 2.4 Data sources and search methods

#### 2.4.1 Search strategy

Comprehensive searches in electronic databases (PUBMED, EMBASE, Cochrane Library, Scopus, AMED, CNKI, and WANGFANG) from inception to present, and other literature sources (such as conference proceedings, and reference lists of included trials). We will not impose any limitations to language and publication time. We will create search strategy sample for PUBMED in Table 1. We will also modify and utilize identical search strategy for other electronic databases.

**Table 1:** Search strategy sample of PUBMED.

Number	Search terms
1	Alzheimer disease
2	Dementia
3	Cognitive impairment
4	Brain disorder
5	Memory problem
6	Depression
7	Mental disorder
8	Psychological problem
9	Emotional response
10	Depressive episode
11	Depressive disorder
12	Or 1–11
13	Sertraline
14	Zoloft
15	4-(3,4-Dichlorophenyl)-3,4-dihydronaphthalene-1(2H)-one
16	4-(3,4-Dichlorophenyl)-1-naphthalenone
17	4-(3,4-Dichlorophenyl)-1-tetralone
18	Or 13-17
19	Randomized
20	Random
21	Randomly
22	Placebo
23	Blind
24	Allocation
25	Control
26	Clinical
27	Trial
28	Study
29	Or 19–28
30	12 and 18 and 29

#### 2.4.2 Study selection

Two team members will independently perform study selection. After searching studies, all citations will be exported to the Endnote X7, and duplicates will be removed. The titles/abstracts found will be read carefully to eliminate relevant studies. Then, all potential articles will be retrieved by full-text to determine eligibility. If there are disagreements between 2 team members, we will consult a third team member to solve them and will reach a final decision. The study scanning and selection process will be summarized in a flow diagram.

#### 2.4.3 Data extraction and management

Two team members will conduct data extraction independently using a predefined data collection form. Any divisions between 2 members will be settled down with a discussion. The extraction sheet consists of title, first author, time of publication, patient information, study setting, study methods, sample size, specifics of intervention and comparator, outcomes, dropouts, follow-up information, adverse events, and conflict of interest.

#### 2.4.4 Dealing with missing data

We will contact primary corresponding author by email or fax to obtain any unclear or missing data. Intention-to-treat analysis will be performed if we cannot obtain those missing or insufficient information.

### 2.5 Risk of bias assessment

The risk of bias of all included trials will be appraised using Cochrane Risk of Bias Tool by 2 independent team members. Each outcome will be assessed through 7 aspects and each item is divided into 3 levels: high, unclear, or low risk of bias. Any conflict between 2 team members will be cleared up by a third team member via discussion.

### 2.6 Statistical analysis

This study will utilize RevMan 5.3 software for data analysis. For dichotomous outcome data, it will be estimated as risk ratio with 95% confidence intervals (CIs). For continuous outcome data, it will be calculated as mean difference and 95% CIs. Heterogeneity among eligible trials will be examined by *I*^2^ statistic. *I*^2^ ≤ 50% is considered as having acceptable heterogeneity, and a fixed-effect model will be employed. Meta-analysis will be carried out if sufficient data on the same outcome are extracted from eligible trials with acceptable heterogeneity. *I*^2^ > 50% is regarded as having obvious heterogeneity, and a random-effect model will be placed. Subgroup analysis will be conducted to explore the possible causes of remarkable heterogeneity. If it is impossible to synthesize the outcome data, a narrative analysis will be utilized to report outcome results.

### 2.7 Subgroup analysis

In case of obvious heterogeneity, we will carry out subgroup analysis in accordance with the different patient characteristics, treatments, and controls.

### 2.8 Sensitivity analysis

We will perform sensitivity analysis to test the robustness and stability of study findings by eliminating low quality and small sample size (less than 10 in each group).

### 2.9 Reporting bias

If sufficient number of eligible trials (over 10 RCTs) are available, we will examine reporting bias using funnel plot and Egger regression test.

## 3 Discussion

Sertraline is commonly utilized to treat depression in patients with AD. Although a variety of studies have suggested that sertraline is an effective therapy for depression after AD, there has been no systematic review presenting evidence of clinical efficacy of sertraline on depression caused by AD. We have organized a protocol for a systematic review of sertraline for the treatment of depression following AD. We will perform comprehensive search in both electronic databases and other literature sources to avoid missing more potential studies. The results of this study may provide robust evidence of sertraline in the treatment of depression caused by AD, which may help both clinical practice and further researches.

## Author contributions

**Conceptualization:** Wei-hua Li, Zhuo-wen Wei, Xiao-feng Liu.

**Data curation:** Wei-hua Li, Xiao-feng Liu.

**Formal analysis:** Zhuo-wen Wei.

**Funding acquisition:** Wei-hua Li.

**Investigation:** Xiao-feng Liu.

**Methodology:** Wei-hua Li, Zhuo-wen Wei.

**Project administration:** Xiao-feng Liu.

**Resources:** Wei-hua Li, Zhuo-wen Wei.

**Software:** Wei-hua Li, Zhuo-wen Wei.

**Supervision:** Xiao-feng Liu.

**Validation:** Wei-hua Li, Zhuo-wen Wei, Xiao-feng Liu.

**Visualization:** Wei-hua Li, Zhuo-wen Wei, Xiao-feng Liu.

**Writing – original draft:** Wei-hua Li, Xiao-feng Liu.

**Writing – review & editing:** Wei-hua Li, Zhuo-wen Wei, Xiao-feng Liu.

## References

[1] Rossor M Alzheimer's disease. BMJ 1993;307:779–82. 8219953 10.1136/bmj.307.6907.779 PMC1696429

[2] Masters CL Beyreuther K Alzheimer's disease. BMJ 1998;316:446–8. 9492674 10.1136/bmj.316.7129.446 PMC2665603

[3] Selko DJ Alzheimer's disease. Cold Spring Harb Perspect Biol 2011;3:a004457. 21576255 10.1101/cshperspect.a004457 PMC3119915

[4] Wenk GL Neuropathologic changes in Alzheimer's disease. J Clin Psychiatry 2003;64: suppl 9: 7–10. 12934968

[5] Jahn H Memory loss in Alzheimer's disease. Dialogues Clin Neurosci 2013;15:445–54. 24459411 10.31887/DCNS.2013.15.4/hjahn PMC3898682

[6] Lin SY Hsu WH Lin CC Association of transfusion with risks of dementia or Alzheimer's disease: a population-based cohort study. Front Psychiatry 2019;10:571. 31474887 10.3389/fpsyt.2019.00571 PMC6706818

[7] Chiu PY Wang CW Tsai CT Depression in dementia with Lewy bodies: a comparison with Alzheimer's disease. PLoS One 2017;12:e0179399. 28617831 10.1371/journal.pone.0179399 PMC5472293

[8] Randall C Mosconi L de Leon M Cerebrospinal fluid biomarkers of Alzheimer's disease in healthy elderly. Front Biosci (Landmark Ed) 2013;18:1150–73. 23747874 10.2741/4170 PMC3904672

[9] Trevisan K Cristina-Pereira R Silva-Amaral D Theories of aging and the prevalence of Alzheimer's disease. Biomed Res Int 2019;2019:9171424. 31317043 10.1155/2019/9171424 PMC6601487

[10] Xia X Jiang Q McDermott J Aging and Alzheimer's disease: comparison and associations from molecular to system level. Aging Cell 2018;17:e12802. 29963744 10.1111/acel.12802 PMC6156542

[11] Yu Q Zhong C Membrane aging as the real culprit of Alzheimer's disease: modification of a hypothesis. Neurosci Bull 2018;34:369–81. 29177767 10.1007/s12264-017-0192-4 PMC5856718

[12] Vandenberghe R Tournoy J Cognitive aging and Alzheimer's disease. Postgrad Med J 2005;81:343–52. 15937198 10.1136/pgmj.2004.028290 PMC1743286

[13] Chow N The practice of filial piety and its impact on long-term care policies for elderly people in Asian Chinese communities. Asian J Gerontol Geriatr 2006;1:31–5.

[14] Yin P Feng X Astellburt T Temporal trends and geographic variations in dementia mortality in China between 2006 and 2012: multilevel evidence from a nationally representative sample. Alzheimer Dis Assoc Disord 2016;30:348–53. 26999577 10.1097/WAD.0000000000000147

[15] Orgeta V Tabet N Nilforooshan R Efficacy of antidepressants for depression in Alzheimer's disease: systematic review and meta-analysis. J Alzheimers Dis 2017;58:725–33. 28505970 10.3233/JAD-161247 PMC5467718

[16] Burke AD Goldfarb D Bollam P Diagnosing and treating depression in patients with Alzheimer's disease. Neurol Ther 2019;8:325–50. 31435870 10.1007/s40120-019-00148-5 PMC6858899

[17] Klöppel S Kotschi M Peter J Separating symptomatic Alzheimer's disease from depression based on structural MRI. J Alzheimers Dis 2018;63:353–63. 29614658 10.3233/JAD-170964 PMC5900555

[18] Yun HM Park KR Kim EC Serotonin 6 receptor controls Alzheimer's disease and depression. Oncotarget 2015;6:26716–28. 26449188 10.18632/oncotarget.5777 PMC4694947

[19] Cassano T Calcagnini S Carbone A Pharmacological treatment of depression in Alzheimer's disease: a challenging task. Front Pharmacol 2019;10:1067. 31611786 10.3389/fphar.2019.01067 PMC6777507

[20] Tu CH MacDonald I Chen YH The effects of acupuncture on glutamatergic neurotransmission in depression, anxiety, schizophrenia, and Alzheimer's disease: a review of the literature. Front Psychiatry 2019;10:14. 30809158 10.3389/fpsyt.2019.00014 PMC6379324

[21] Narita Z Yokoi Y Transcranial direct current stimulation for depression in Alzheimer's disease: study protocol for a randomized controlled trial. Trials 2017;18:285. 28629447 10.1186/s13063-017-2019-z PMC5477338

[22] Longmire CVF Drye LT Frangakis CE Is sertraline treatment or depression remission in depressed Alzheimer patients associated with improved caregiver well being? Depression in Alzheimer's Disease Study 2. Am J Geriatr Psychiatry 2014;22:14–24. 24314887 10.1016/j.jagp.2013.02.014 PMC3910508

[23] Takemoto M Ohta Y Hishikawa N The Efficacy of sertraline, escitalopram, and nicergoline in the treatment of depression and apathy in Alzheimer's disease: the Okayama Depression and Apathy Project (ODAP). J Alzheimers Dis 2020;76:769–72. 32568205 10.3233/JAD-200247

[24] Lyketsos CG Sheppard JM Steele CD Randomized, placebo-controlled, double-blind clinical trial of sertraline in the treatment of depression complicating Alzheimer's disease: initial results from the Depression in Alzheimer's Disease study. Am J Psychiatry 2000;157:1686–9. 11007727 10.1176/appi.ajp.157.10.1686

[25] Magai C Kennedy G Cohen CI controlled clinical trial of sertraline in the treatment of depression in nursing home patients with late-stage Alzheimer's disease. Am J Geriatr Psychiatry 2000;8:66–74. 10648297 10.1097/00019442-200002000-00009

[26] Volicer L Rheaume Y Cyr D Treatment of depression in advanced Alzheimer's disease using sertraline. J Geriatr Psychiatry Neurol 1994;7:227–9. 7826491 10.1177/089198879400700406

[27] Shamseer L Moher D Clarke M PRISMA-P Group. Preferred reporting items for systematic review and meta-analysis protocols (PRISMA-P) 2015: elaboration and explanation. BMJ 2015;350:g7647. 25555855 10.1136/bmj.g7647

